# Resistance to Cucumber Green Mottle Mosaic Virus in *Cucumis melo*

**DOI:** 10.3390/plants10061077

**Published:** 2021-05-27

**Authors:** Leticia Ruiz, Carmelo López, Belén Picó, Dirk Janssen

**Affiliations:** 1 IFAPA, Centro La Mojonera, 04745 La Mojonera, Spain; mleticia.ruiz@juntadeandalucia.es; 2 Institute for the Conservation and Breeding of Agricultural Biodiversity (COMAV), Universitat Politècnica de València, Camino de Vera s/n, 46022 Valencia, Spain; clopez@upv.es (C.L.); mpicosi@btc.upv.es (B.P.)

**Keywords:** real-time RT-PCR, viral load, CGMMV, tobamovirus

## Abstract

Cucumber green mottle mosaic virus (CGMMV) is a severe threat to melon production worldwide. At present, there are no cultivars available on the market which show an effective resistance or tolerance to CGMMV infection; only wild *Cucumis* species were reported as resistant. Germplasm accessions of *Cucumis melo*, as well as *C. anguria*, *C. ficifolius*, *C. myriocarpus* and *C. metuliferus,* were mechanically infected with isolates belonging to the European and Asian strain of CGMMV and screened for resistance by scoring symptom severity and comparing the accumulation of virus by qRT-PCR. The wild species *C. anguria* and *C. ficifolius* showed no symptoms and did not accumulate CGGMV following inoculation, while *C. metuliferus* was highly susceptible to the isolates of both strains of CGMMV. The virus accumulated also in *C. myriocarpus* and the European isolate produced symptoms, but the Asian isolate did not. Thirty *C. melo* accessions were susceptible to CGMMV. An isolate-dependent expression of symptoms was observed in 16 melon accessions: they showed mild and severe symptoms at 14 and 21 days after inoculation with the European and Asian isolate, respectively. Freeman’s Cucumber showed few or no symptoms following inoculation with the isolate of either CGMMV strain. This particular accession also showed reduced virus accumulation, whereas most other tested germplasm accessions showed significantly higher viral loads and, therefore, may well be a candidate for breeding programs aiming to reduce the losses produced by CGMMV with resistant commercial melon cultivars.

## 1. Introduction

Diseases produced by viruses cause economic losses in commercial cucurbit production around the world [1]. Among these viruses, cucumber green mottle mosaic virus (CGMMV) represents a major risk to the production of melons, watermelons and cucumbers. CGMMV belongs to the genus *Tobamovirus*, family *Virgaviridae* [2], and causes systemic mottle and mosaic symptoms on the leaves of cucurbitaceous plants. In susceptible watermelon genotypes, the virus causes a pulp deterioration called blood flesh disease and the fruit loses its marketable value [3]. In cucumbers, the virus causes deformation and mosaic symptoms [4]. In melons, young leaves develop initial mottle and mosaic symptoms that often disappear from mature foliage. Their fruits develop different degrees of malformation, mottling and surface netting [5]. It is mechanically, pollen- and seed-transmitted [6].

CGMMV was first described infecting cucumbers in England in 1935 by Ainsworth [7]. Its incidence in other countries of the world has increased rapidly during the last decade, possibly through the international seed trade following cucurbit seed crop production in tropical or subtropical countries [6]. Moreover, seed-testing routines for CGMMV may be inadequate, which allows for the rapid and worldwide spread of the virus. Contaminated seeds provide a route for the movement of the virus between countries and its introduction into new areas, and several seed treatments currently used were found to be insufficient for eliminating the virus from contaminated seed lots. In addition, like other tobamoviruses, CGMMV can survive for a long time on plant debris from infected crops [8]. Therefore, control depends on early monitoring, awareness of the farmers and appropriate crop management, but even implementing these measures does not guarantee success [9].

Worldwide, CGMMV isolates are grouped in two major clusters based on biological differences and genome sequences: a first cluster constitutes the European strain and includes most isolates from France, the Netherlands and Uzbekistan. A second cluster is formed by isolates from Asian countries, such as Japan and South Korea. Spain is currently the first country where both these strains have been described co-infecting the same crops and in the same region [10]. Although isolates of both strains differ in their genome sequences, they do not differ in terms of the systemic symptoms expressed on leaves of infected cucumbers. However, they behave differently in *Chenopodium amaranticolor*, on which local lesions appear only when isolates of the Asian strain are inoculated [10].

Commercial tomato and pepper hybrids that carry virus-resistant genes successfully control tobamoviruses in solanaceous crops. Resistance is an important factor that determines concentrations of virus in several virus-cucurbits host plant pathosystems. This happens, for example, with cucurbit yellow stunting disorder virus and Watermelon mosaic virus in melon [11,12,13]. However, in the case of CGMMV, there is an urgent need for resistant melon cultivars that have restricted movement and replication of virus as well as reduced symptom development. Melon is an important crop, of which about 150 million tons of fruit are produced worldwide, including 66,000 tons in Spain during 2018 [14]. However, commercial melon varieties resistant to CGMMV are currently unavailable. Efforts have been made to produce transgenic resistance in melon [15]. The development of resistant varieties through conventional breeding could offer a good solution to this disease, which continues to escalate.

Rajamony et al., screened 187 accessions of *C. melo* and eight wild *Cucumis* species. Six wild species were found to be resistant (*C. africanus*, *C. ficifolius*, *C. figarei*, *C. meeussii*, *C. myriocarpus* and *C. zeyheri*) but only a few accessions of the Indian momordica and Kachri melon groups displayed a partially resistant response, with recessive and polygenic control [16,17,18]. Others confirmed the behavior as symptomless carriers of momordica accessions and the immune response of *C. figarei* and *C. zeyheri* [19]. Apart from these melon types, new resistance was reported in a Korean accession of the oriental *makuwa* group [20]. Resistance was partial and temperature-dependent, and controlled by two independent complementary recessive genes [21]. These previous studies used isolates from the Asian type of CGMMV. Thus, no full resistance to the virus has been reported yet in melon.

In the present paper we inoculated isolates belonging to the European and the Asian strain of CGMMV in a collection of 47 accessions of *C. melo*, selected to represent the variability of the species, and four wild *Cucumis* species: *C. anguria, C. ficifolius, C. metuliferus* and *C. myriocarpus.* We report on the evolution of symptom expression for both virus isolates in all accessions, and that of the viral loads on a representative number of accessions.

## 2. Results

### 2.1. Symptom Expression

**Figure 1:**
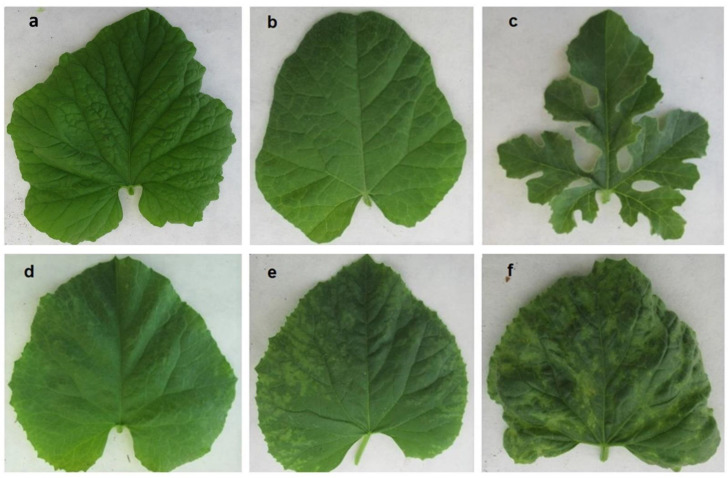
Range of symptoms of CGMMV in *Cucumis* at 15 days following inoculation with CGMMV-SP: (**a**) “0” in Freeman’s Cucumber, (**b**) in *C. ficifolius*, (**c**) in *C. myriocarpus*, (**d**) “1” in PMR-45, (**e**) “2” in PI 125951 and (**f**) “3” in PI 314427, as representative *C. melo* accessions.

Single inoculations in melon accessions with CGMMV isolates CGMMV-SP and CG-SPCu16 that represent the European and the Asian strains, respectively, produced symptoms that were scored according to the scales from 0 to 3, as defined in the Materials and Methods (Figure 1).

The time of appearance of these symptoms in individual plants was variable, but most of the replicate plants of each accession showed similar symptoms at 21 dpi (Table S1). The plants were observed beyond that time, but no change in the symptoms was spotted. Severe symptoms (score 3) were found following inoculation with both isolates in 24 *C. melo* accessions which belonged to the Asian ameri and the Spanish *ibericus* types, such as Birjucekutskaja and Piñoncillo, but also to the Asian Dudaim types, Indian Kachri and momordica and African and Indian wild *agrestis*, as well as to oriental conomon melons.

Some melon accessions also showed severe symptoms against CG-SPCu16, but intermediate symptoms with CGMMV-SP (the Spanish *ibericus* Rochet accession BGV004884 and the *makuwa* accession PI 420176 from Japan) while two others showed intermediate symptoms with CG-SPCu16 and severe symptoms with the European isolate (the oriental *chinensis* accession PI 161375 and the *flexuosus* melon BGV004853). Two additional melon accessions were also susceptible, but the Indian momordica PI 381789 displayed intermediate symptoms with both virus isolates, while the Khira melon from India PI 271332 had a variable response to the virus isolate CG-SPCu16. A virus isolate-dependent expression of symptoms was observed in 16 melon accessions: they showed mild and severe symptoms at 14 and 21 dpi when infected with the European and Asian isolate, respectively (many ameri, chandalak or casaba types from Eastern Europe, Asia Minor, Central Asia and Northern Africa and the *cantalupensis* and *reticulatus* groups, such as the Turkish Kirkagac accession PI 169305, the Georgian Nanatri melon or the *cantalupensis* Dvash Ha Ogen). The only accession resistant to both isolates of the virus was the conomon Japanese accession Freeman’s Cucumber. Regarding wild types, *C. metuliferus* expressed severe symptoms with the isolates of both CGMMV strains, and *C. myriocarpus* displayed intermediate symptoms following the inoculation with CGMMV-SP and none with CG-SPCu16. The inoculation with CGMMV produced no symptoms in *C. anguria, and C. ficifolius* (Table S1).

### 2.2. CGMMV Viral Loads

Plants inoculated with CGMMV had relative viral loads that ranged from undetected to values of 3.16e^9^ (BGV015753 at 14 dpi SP) (Table S2).

**Figure 2:**
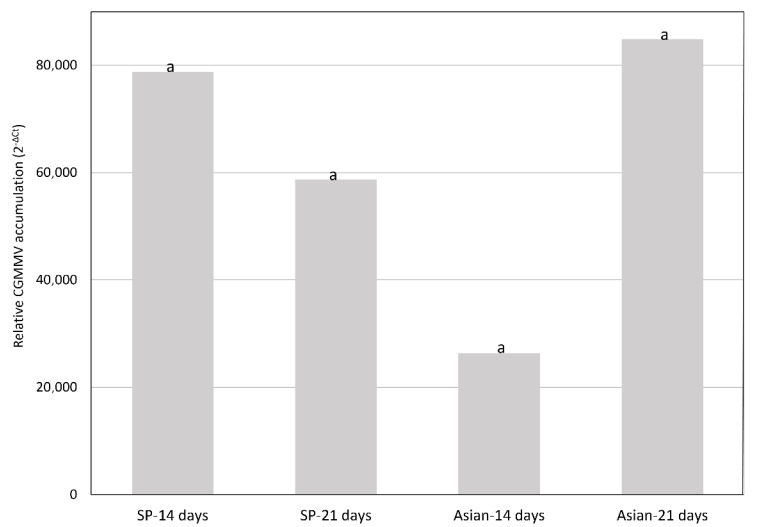
Mean of relative cucumber green mottle mosaic virus (CGMMV) accumulation (calculated as 2^−ΔCt^) at 14 and 21 days following inoculation in plants susceptible to the European (CGMMV-SP) and Asian (CG-SPCu16) isolates (*n* = 30). Letters show no significant differences were found between the variables (α = 0.05, Kruskal–Wallis test).

**Figure 3:**
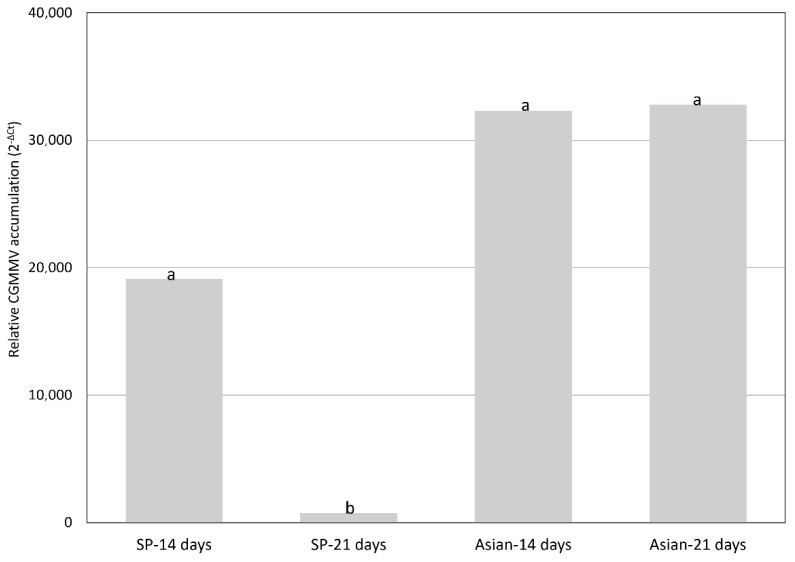
Mean of relative cucumber green mottle mosaic virus (CGMMV) RNA accumulation (calculated as 2^−ΔCt^) at 14 and 21 days following inoculation in plants resistant to the European (CGMMV-SP) and susceptible to the Asian isolates (CG-SPCu16) (*n* = 16). Letters show significant differences found between the variables (α = 0.05, Kruskal–Wallis test).

**Figure 4:**
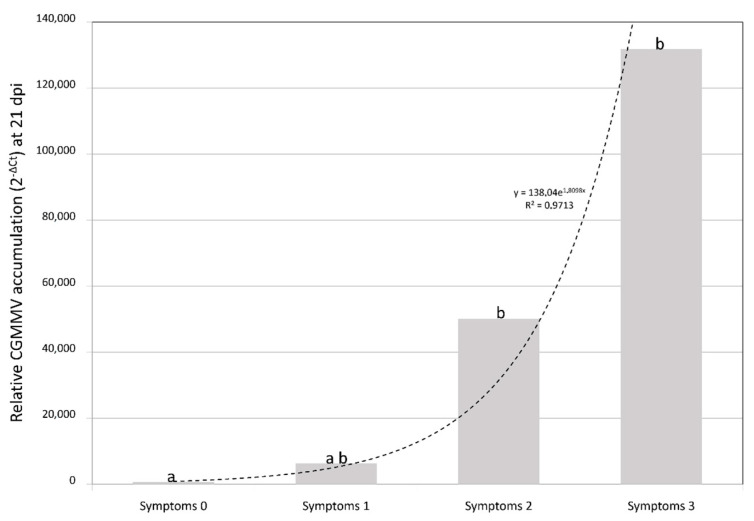
Mean of relative cucumber green mottle mosaic virus (CGMMV) accumulation (calculated as 2^–ΔCt^) in plants within symptomatic classes 0 (*n* = 16), 1 (*n* = 2), 2 (*n* = 9) and 3 (*n* = 67). The dotted line represents the exponential relationship between both variables. Letters show significant difference found between the variables (α = 0.05, Kruskal–Wallis test).

Among the wild *Cucumis* species, *C. anguria* and *C. ficifolius* tested negative for CGMMV, whereas CGMMV concentrations in *C. metuliferus* and *C. myriocarpus* ranged between the orders of e^5^ to e^7^ with the CGMMV-SP, and e^3^ to e^4^ with CG-SPCu16. Melon accessions that showed severe symptoms with both virus isolates had similar viral loads at 14 and 21 dpi (Figure 2). However, the European isolate accumulated less than the Asian isolate at 21 dpi in accessions in which it produced milder symptoms when compared with the Asian isolate (*p* < 0.05) (Figure 3). Freeman’s Cucumber also accumulated smaller amounts of virus (maximum viral load value 7.44e^2^ at 14 dpi with CGMMV-SP), and CGMMV was not detected at 21 dpi in plants inoculated with CG-SPCu16 (Table S2). The average viral loads correlated with the expression of symptoms (Figure 4).

## 3. Discussion

We tested a collection of 47 melon accessions for resistance to CGMMV. The majority of genotypes were very susceptible after mechanical inoculation, as was made clear by the expression of typical CGMMV symptoms and the accumulation of virus in apical leaves. The high susceptibility observed along the whole range of diversity of this species confirmed that this virus represents a major threat to melon cultivation. The majority of the accessions tested were susceptible to the European (CGMMV-SP) and the Asian (CG-SPCu16) isolates of CGMMV, which is relevant to countries such as Spain, where both strains occur, sometimes even in the same greenhouse [10] Among these accessions were Spanish landraces, which may have been as expected, since they do not usually have resistances to viruses. Interestingly, within the Spanish accessions, two, Rochet (BGV004884) and Alficos (BGV004853), showed a milder response to the European and Asian isolates of CGMMV, respectively, which is relevant in view of the recovery of its cultivation in Spain [22].

Equally susceptible as most Spanish landraces were also most of the accessions of Asian origin that belong to many melon groups such as ameri, Dudaim, even certain wild types (wild *agrestis* accessions PI 164797, PI 185111 or PI 536476) and Kachri (PI 614521), groups, where resistance had been previously described. Other melon accessions that were susceptible to both main virus isolates were some Indian accessions of momordica and conomon that otherwise are multi-resistant to other viruses: Shiro Uri Okayama, which is resistant to cucumber mosaic virus (CMV) [23], accession PI 124112, which is resistant to cucurbit aphid-born yellows virus and tomato leaf curl New Delhi virus [13] and momordica PI 180280, which is resistant to papaya ringspot virus and other potyviruses [17,24,25]. Rajamony et al., found mild symptoms when they inoculated Kachri melons [16]. In our study, PI 164493 was found susceptible to CGMMV but strong symptoms took one additional week to appear when inoculated with the Asian isolate than with the European one (Table S1). Although plants were observed for symptom expression beyond 21 dpi, no further changes were found, agreeing with the evolution of CGMMV in cucumber [26].

The expression of symptoms following inoculation with CGMMV was variable in other melon accessions. The makuwa melon PI 420176 already expressed severe symptoms with the Asian isolate after 14 dpi, and intermediate symptoms with the European isolate after 21 dpi, similarly to the Rochet type. The *chinensis* Korean accession PI 161375 “Sonwang Charmi” and the *flexuosus* melon BGV004853 showed the opposite behavior, expressing strong symptoms with the European isolate after 21 dpi, and intermediate symptoms with the Asian isolate after 14 dpi. PI 420176 and PI 161375 are resistant to CMV and multiresistant, respectively [13,27]. The resistance to CMV, however, is dependent on the strain of the virus, which constitutes another example of the importance of considering different viral strains in the characterization of resistance in plants [28]. Accession PI 161375 is also resistant to a different cucurbit-infecting tobamovirus, Kyuri green mottle mosaic virus [29], and whether the isolate-specific intermediate resistance that we observed suggests a common origin of resistance directed towards Asian cucurbit infecting tobamoviruses could be further investigated.

The resistance of the other 16 melon accessions depended on the isolate of CGMMV. They were very susceptible to the Asian isolate but showed resistance to the European isolate. These included mostly accessions belonging to the *cantalupensis* and *reticulatus* (Europe) and ameri, chandalak and casaba (Asia and North-Africa) melon types (Table S2).

The clear identification of CGMMV isolates and strains is fundamental to determine resistance to CGMMV, as has already been reported in cucumber [26]. Here, we confirmed that *C. anguria* is resistant to isolates of both the European and Asian strains [26]. Others have found this plant species to be resistant to the European [30], and susceptible to the Asian strains of CGMMV [16]. *C. myriocarpus* produced intermediate symptoms with the European isolate and remained symptomless following the inoculation with the Asian strain, whereas *C. ficifolius* was symptomless with the isolates of both strains (Table S1). Earlier studies found *C. myriocarpus* and *C. ficifolius* symptomless to the Asian isolate of CGMMV [16].

The Asian isolate of CGMMV proved to be very aggressive on many of the varieties tested. Interestingly, we found resistance to the European isolate in a significant number of *cantalupensis*, *reticulatus*, ameri, chandalak, cand Kachri accessions, which originate from European, Central Asia, Asia Minor and North African countries. Asian ameri and chandalak types seem to be at the origin of the *cantalupensis* varieties which are molecularly different from far eastern melon varieties [31,32,33], and which would fit with a common response to the virus. These accessions are interesting candidates for melon breeding for resistance to the European CGMMV strain. The highest degree of resistance to both CGMMV isolates was found for Freeman’s Cucumber; this conomon melon type from Japan is also resistant to CMV [34]. The genetics of resistance to CGMMV in melon is not known. CMV and CGMMV are both linear positive-sense RNA viruses but they are unrelated. CMV is a member of the family *Bromoviridae*, genus *Cucumovirus*. This virus has a very wide host range and is predominantly transmitted from plant to plant by aphids in a stylet-borne fashion. Its genome is tripartite surrounded by isometric particles. On the other hand, CGMMV is member species of a different plant virus family and, although, at least 15 weed species from nine different plant families have been identified as potential natural hosts, its host range is restricted to plant species in the family *Cucurbitaceae*. It is not transmitted by insects, but mainly through contact and infected soil or seeds [5].

Here, we report on melon accessions that are described for the first time as resistant to CGMMV. Freeman’s Cucumber (Conomon, Japan) is shown here, for the first time, to be resistant to CGMMV, and this entry is of enormous interest as it crosses perfectly with melons. With respect to wild species, we confirm the resistance of *C. anguria* and *C. ficifolius* to isolates of both CGMMV strains. These species do not cross with melons so they cannot be used as sources of resistance in breeding programs, but they are useful as rootstocks. In fact, a hybrid rootstock between *C. anguria* and *C. ficifolius* for melon grafting has already been described [22,35]. This rootstock has resistance to nematodes and soil fungi, but the fact that it also has resistance to CGMMV, a virus that is also transmitted through the soil, makes it much more interesting.

## 4. Materials and Methods

### 4.1. Plant Material

Forty-seven accessions were selected from a previously established melon core collection to represent some of the most important *C. melo* groups, including accessions of the momordica, Kachri, and makuwa, the melon groups in which CGMMV has been previously reported, and additional *inodorus-ibericus*, *cantalupensis-reticulatus*, casaba, Asian ameri-chandalack, *flexuosus*, Dudaim, conomon, *chinensis* and wild *agrestis* types not screened previously [17,31,32,36]. Accessions of this collection come from different germplasm and breeder collections, and were maintained and multiplied under insect-proof conditions by the *Cucurbits* breeding group at COMAV-UPV. The type and origin of these accessions is shown in Table 1. Four wild species C. *anguria, C. ficifolius, C. myriocarpus* and *C. metuliferus* coming from the COMAVs germplasm collection were also included.

### 4.2. Virus Sources for Mechanical Inoculation

In order to investigate differences in resistance, we mechanically inoculated plants with each one of two isolates that represent the two different strains of CGMMV: the strain of European origin (CGMMV-SP) (GenBank GQ411361) and that of Asian origin (CG-SPCu16), as described by [10]. Before its use in screening the *Cucumis* accessions, the isolates were propagated after mechanical inoculation in cucumber (cv. Cumlaude) in a plant growth chamber. Infected plants were grown in an insect-proof greenhouse where temperature was partially controlled (25–30 °C). Approximately 3 weeks after inoculation, the leaves of plants that showed typical symptoms of virus infection were used as the virus source [26].

### 4.3. Mechanical Inoculation

For each virus isolate, 0.5 g of leaf tissue was taken at 5 weeks after sowing and from the second leaf down from the plant apex which displayed CGMMV symptoms. The tissue was homogenized in 1.5 mL of 50 mM sodium phosphate buffer (pH 7.0) and inoculated mechanically by rubbing 150 µL of the extract onto leaves dusted with carborundum powder. At least ten plants of each accession were inoculated with each virus isolate, and two plants with sodium phosphate buffer as controls on 7th of October 2019. All inoculated plants were maintained in an insect-proof greenhouse under controlled conditions.

### 4.4. Evaluation of Symptoms and Quantification of the Virus

Inoculated plants were evaluated for the expression of CGMMV symptoms at 14 and 21 days post inoculation (dpi) using the following scale: 0 (symptomless), 1 (mild symptoms, as initial mottle mosaic on leaves), 2 (intermediate symptoms, as evident leaf mottle mosaic on leaves) and 3 (severe symptoms as mottle mosaic, interveinal chlorosis and blistering on leaves) (Figure 1). The presence of the virus was analyzed in two pools of five plants each. At 14 and 21 dpi, samples of 0.2 g tissue were removed from the second leaf (not inoculated, and possibly representing systemic infection) from the apex. The pooled samples were ground to a fine powder in liquid nitrogen in a pestle and mortar and placed in a sterile microcentrifuge tube. Total RNA was extracted with Trizol reagent (Invitrogen). The resulting pellet was resuspended in 50 µL DEPC-treated water and stored at −80 °C. RNA was quantified with an ND-2000c spectrophotometer (NanoDrop Technologies, Thermo Fisher Scientific S.L, Madrid, Spain) and diluted to a final concentration of 50 ng/µL. Real-time RT-PCR reactions were set up in 96-well reaction plates: one microliter aliquots were used as templates in the RT-PCR reactions of 20 µL, containing 10 µL Master Mix (qMAXSen™ One-Step Green RT-qPCR Kit, Canvax Biotech, Cordoba, Spain), 1 µL forward primer, 1 µL reverse primer, 1 µL total RNA and 7 µL DEPC-water. The primers for CGMMV were 5′-GCATAGTGCTTTCCCGTTCAC-3′ (sense) at positions 6285-6305 nt and TGCAGAATTACTGCCCATAGAAAC-3′ (antisense) at positions 6362-6385 nt of the genome sequence, as described [25]. Primers and probe for the internal control were designed based on the peptidyl-prolyl cis-trans isomerase, cyclophilin (CYP 7) gene MELO3C025848.2 [37]: primers CYP-7F (5′-TACTGGACCAGGCCTCCTAT-3′), CYP-7R (5′-TAGGTGTTCCGTTCTGCCTT-3′) and Taqman probe CYP-7TM (5′-TGGCAAACGCTGGTCCAGACACCA-3′)**.** Cycling conditions consisted of incubation at 95 °C for 5 min, 45 cycles of 95 °C for 5 s and 60 °C for 30 s. Relative CGMMV levels were calculated using the 2^−ΔCt^ expression, a variation of the Livak method [38,39], where ratio (reference/target) = 2^−ΔCt^ = 2^−(Ct (viral target)−Ct (reference gen))^. Three technical replications were performed per sample and the tests were run on an ABI Prism 7000 DNA sequence detection system (Thermo Fisher Scientific S.L, Madrid, Spain) as followed: 10 min at 50 °C, 1 min at 95 °C, 40 cycles of 15 s at 95 °C and 1 min at 60 °C.

### 4.5. Data Analysis

Resistance was evaluated as the response of the host plant to virus infection, estimated from symptom severity and the viral titers in all inoculated plants. The interaction effects from inoculated isolates of virus, and dpi on viral loads, were investigated using the Kruskal–Wallis test. All effects were tested at the 5% significance level. Analyses were performed using Statistics 9.1 statistical software.

## Figures and Tables

**Table 1:** Melon and wild *Cucumis* accessions screened for resistance to CGMMV.

Accession ID	Accession Name	Country of Origin
***C. melo* subsp. *Melo***		
*Inodorus-ibericus Spanish landraces*		
BGV016451	Amarillo Groc	Spain
BGV015753	Blanco	Spain
BGV004871	Tendral	Spain
BGV013188	Pipa de oro	Spain
BGV003686	Piñoncillo	Spain
BGV003692	Blanco Redondo	Spain
BGV003718	Mochuelo	Spain
BGV004884	Melón Rochet	Spain
*Ameri/adana/chandalak/casaba and other landraces from Europe and Asia*		
PI 125951	3584	Afghanistan
BGV001367	Nanatri	Georgia
PI 314427	Koljoznitza	Georgia
CUM 259	Apelsinaja	Russia
PI 276660	VIR610-*chandalak*	Afghanistan
BGV001364	Asli	Tunisia
BGV001365	Tokash	Tajikistan
PI 169331	Altimbas	Turkey
PI 506459	Salgirskaja	Ukraine
BGV001362	Kizil-uruk	Uzbekistan
PI 169305	Kirkagac	Turkey
PI 476342	Imljskaha	Kazakhstan
Am-BirUkr	Birjucekutskaja	Ukraine
In-HamiChi	Hami Melon	China
Am-OuzUzb2	Ouzbeque	Uzbekistan
Am-SouiMor	Souilah	Morocco
*Cantalupensis and reticulatus commercial cultivars and landraces* *from Europe, Asia, and America*		
Can-VedFran	Vedrantais	France
Can-NYIsr	Noy Israel	Israel
Can-NOFran	Nantais Oblong	France
Ames 26811	PMR-45	USA
La-OgenBul	Dvash Ha Ogen	Bulgaria
*Flexuosus and Dudaim accessions*		
BGV004853	Alficoz	Spain
PI 273438	Queen Anne’s Pocket Melon	Georgia
***C. melo* subsp. *agrestis***		
*Momordica accessions and other landraces from India*		
PI 124112	2564	India
PI 180280	Kahkri	India
PI 381781	Sm1	India
PI 381789	Sm9	India
PI 271332	Khira	India
*Conomon, chinensis and makuwa accessions from Far East*		
PI 420176	Ginsen makuwa	Japan
Con-FreeCJa	Freeman’s Cucumber	Japan
PI 161375	Songwhan Charmi	Korea
Con-ShiroJa	Shiro Uri Okayama	Japan
*Kachri and wild agrestis from Asia and Africa*		
Am-SarakIran	Sarakhs	Iran
PI 614521	KSM 531 Kachri	India
PI 164493	Kakru	India
PI 185111	15591	Ghana
PI 164797	9227	India
PI 532839	Chibbar	India
PI 536476	KLM 1733	Maldives
**Wild *Cucumis* spp.**		
BGV11135	*C. metuliferus*	
BGV012786	*C. ficifolius*	
BGV012795	*C. anguria*	
BGV008535	*C. myriocarpus*	

## Data Availability

All data generated or analyzed are contained within the present article.

## Supplementary Materials

The following are available online at https://www.mdpi.com/article/10.3390/plants10061077/s1, Table S1. Symptom expression in *C. melo* and four wild *Cucumis* species inoculated with the European and Asian isolates of CGMMV; Table S2. Relative accumulation (RA) and standard error (SE) of CGMMV in *C. melo* and in four wild *Cucumis* species inoculated with the European and Asian isolates.
