# Supplementary material for: Resistance to Cucumber Green Mottle Mosaic Virus in Cucumis melo
Source: Plants (Basel). 2021 May 27;10(6):1077. doi: 10.3390/plants10061077 (PMC8227257; doi:10.3390/plants10061077)
Supplement: Supplementary file 1 [file plants-10-01077-s001.zip › plants-1195471-supplementary.pdf]

plants

Supplementary Materials

Table S1. Symptom expression in *C. melo* and four wild *Cucumis* species inoculated with the European and Asian isolate of CGMMV.

	European						Asian					
	Symptomatic Plants (%) *		Symptom Severity **				Symptomatic Plants (%)		Symptom Severity			
**Accession, Type, and Country of Origin**	**14 dpi**	**21 dpi**	**14 dpi**		**21 dpi**		**14 dpi**	**21 dpi**	**14 dpi**		**21 dpi**	
BGV004884, Rochet,Ibericus, Spain	0	100	0,0	0-0	2,0	2-2	100	100	1,0	1-1	3,0	3-3
PI 420176 (Ginsen makuwa) Makuwa, Japan	0	100	0,0	0-0	2,0	2-2	100	100	3,0	3-3	3,0	3-3
Birjucekutskaja * (Am-BirUkr) Ameri, Ukraine	100	100	3,0	3-3	3,0	3-3	100	100	3,0	3-3	3,0	3-3
PI 314427 (Koljoznitza) Ameri, Georgia	100	100	3,0	3-3	3,0	3-3	100	100	3,0	3-3	3,0	3-3
PI 185111 (15591) Wild Agrestis, Ghana	100	100	2,0	2-2	3,0	3-3	100	100	3,0	3-3	3,0	3-3
Ouzbeque * (Am-OuzUzb2) Ameri, Uzbekistan	100	100	3,0	3-3	3,0	3-3	100	100	2,0	2-2	3,0	3-3
PI 161375 (Songwhan Charmi) Chinensis, Korea	0	100	0,0	0-0	3,0	3-3	100	100	2,0	2-2	2,0	2-2
PI 273438 (Queen Annes Pocket Melon) Dudaim, Georgia	100	100	3,0	3-3	3,0	3-3	100	100	3,0	3-3	3,0	3-3
Shiro Uri Okayama * (Con-ShiroJa) Conomon, Japan	0	100	0,0	0-0	3,0	3-3	0	100	0,0	0-0	3,0	3-3
PI 124112 (2564) Momordica, India	100	100	3,0	3-3	3,0	3-3	100	100	3,0	3-3	3,0	3-3
BGV 016451 (Amarillo Groc) Ibericus, Spain	100	100	3,0	3-3	3,0	3-3	100	100	3,0	3-3	3,0	3-3
BGV015753 (Blanco) Ibericus, Spain	100	100	3,0	3-3	3,0	3-3	100	100	3,0	3-3	3,0	3-3
BGV013188 (Pipa de Oro) Ibericus, Spain	100	100	3,0	3-3	3,0	3-3	100	100	3,0	3-3	3,0	3-3
Asli (In-AsliTun) Ameri Tunisia	100	100	3,0	3-3	3,0	3-3	100	100	3,0	3-3	3,0	3-3
BGV003686 (Piñoncillo) Ibericus, Spain	100	100	3,0	3-3	3,0	3-3	100	100	3,0	3-3	3,0	3-3
BGV003692 (Blanco Redondo) Ibericus, Spain	100	100	3,0	3-3	3,0	3-3	100	100	3,0	3-3	3,0	3-3
BGV003718 (Mochuelo) Ibericus, Spain	100	100	3,0	3-3	3,0	3-3	100	100	3,0	3-3	3,0	3-3
BGV001365 (Tokash) Ameri, Tajikistan	67	100	1,3	0-2	3,0	3-3	100	100	3,0	3-3	3,0	3-3
BGV001632 (Kizil-uruk) Ameri, Uzbekistan	100	100	3,0	3-3	3,0	3-3	100	100	3,0	3-3	3,0	3-3
BGV004871 (Tendral) Ibericus, Spain	100	100	3,0	3-3	3,0	3-3	100	100	3,0	3-3	3,0	3-3
PI 164493, kakru, Kachri, India	100	100	3,0	3-3	3,0	3-3	0	100	0,0	0-0	3,0	3-3

PI 164797, 9227, wild agrestis, India	100	100	3,0	3-3	3,0	3-3	100	100	3,0	3-3	3,0	3-3
PI 180280, Kahkri, Momordica, India	100	100	3,0	3-3	3,0	3-3	100	100	3,0	3-3	3,0	3-3
PI 381781, Sm1, Momordica, India	100	100	3,0	3-3	3,0	3-3	100	100	3,0	3-3	3,0	3-3
PI 532839, chibbar, Wild agrestis, India	100	100	3,0	3-3	3,0	3-3	100	100	3,0	3-3	3,0	3-3
PI 536476, KLM 1733, Maldives	100	100	3,0	3-3	3,0	3-3	100	100	3,0	3-3	3,0	3-3
PI 614521, KSM 531, Kachri, India	100	100	3,0	3-3	3,0	3-3	100	100	3,0	3-3	3,0	3-3
PI 271332, Khira, Landrace, India	100	100	1,0	1-1	3,0	3-3	56	78	1,7	0-3	2,3	0-3
BGV004853, Alficos, Flexuosus, Spain	100	100	1,0	1-1	3,0	3-3	100	100	2,0	2-2	2,0	2-2
PI 381789 (Sm9) Momordica, India	100	100	1,0	1-1	2,0	2-2	100	100	1,0	1-1	2,0	2-2
PI 169305, Kirkagac, cassaba, Turkey	0	0	0,0	0-0	0,0	0-0	100	100	3,0	3-3	3,0	3-3
PI 476342, Imljskaha, Ameri, Kazakhstan	100	0	2,0	2-2	0,0	0-0	100	100	3,0	3-3	3,0	3-3
BGV001367, Nanatri, Ameri, Georgia	0	0	0,0	0-0	0,0	0-0	100	100	3,0	3-3	3,0	3-3
La-OgenBul, Dvash Ha Ogen, Cantalupensis, Bulgaria	0	0	0,0	0-0	0,0	0-0	100	100	3,0	3-3	3,0	3-3
In-HamiChi, HamiMelon, Landrace, China	80	0	1,6	0-2	0,0	0-0	100	100	3,0	3-3	3,0	3-3
Can-NOFran , Nantais Oblong, Cantalupensis, France	0	0	0,0	0-0	0,0	0-0	100	100	3,0	3-3	3,0	3-3
Can-NYIsr, Noy Israel, Cantalupensis, Israel	0	0	0,0	0-0	0,0	0-0	100	100	3,0	3-3	3,0	3-3
Ames 26811, PMR-45, Reticulatus, USA	33	56	0,3	0-1	0,6	0-1	100	100	3,0	3-3	3,0	3-3
Am-SarakIran , Sarakhs, Kachri, Iran	0	0	0,0	0-0	0,0	0-0	100	100	2,0	2-2	2,0	2-2
Am-SouiMor, Souilah, Landrace, Morocco	100	0	1,0	1-1	0,0	0-0	100	100	2,0	2-2	3,0	3-3
PI 125951, 3584, Ameri, Afghanistan	100	0	2,0	2-2	0,0	0-0	100	100	3,0	3-3	3,0	3-3
Can-VedFran, Vedrantais, Cantalupenis, France	0	0	0,0	0-0	0,0	0-0	100	100	2,2	2-3	2,0	2-2
CUM 259, Apelsinaja, Ameri, Russia	0	0	0,0	0-0	0,0	0-0	100	100	3,0	3-3	3,0	3-3
PI 276660, VIR610-Chandalak, Chandalak, Afghanistan	0	0	0,0	0-0	0,0	0-0	100	100	2,0	2-2	3,0	3-3
PI 169331, Altimbas, Ameri, Turkey	0	0	0,0	0-0	0,0	0-0	100	100	3,0	3-3	3,0	3-3
PI 506459, Salgirskaja Ameri, Ukraine	0	0	0,0	0-0	0,0	0-0	100	100	3,0	3-3	3,0	3-3
Freeman's Cucumber (Con-FreeCJa) Conomon, Japan	0	0	0,0	0-0	0,0	0-0	0	100	0,0	0-0	1,0	1-1
BGV012795, *C. anguria*	0	0	0,0	0-0	0,0	0-0	0	0	0,0	0-0	0,0	0-0
BGV012786, *C. ficifolius*	0	0	0,0	0-0	0,0	0-0	0	0	0,0	0-0	0,0	0-0
BGV011135, *C.metuliferus*	100	100	1,0	1-1	3,0	3-3	0	100	0,0	0-0	3,0	3-3
BGV008535, *C.myriocarpus*	0	100	0,0	0-0	2,0	2-2	0	0	0,0	0-0	0,0	0-0

* Percentages from 10 inoculated plants; **Symptom severity at 14 and 21 days post inoculation (dpi). Average level and range of disease in each selected accession (0, Resistant; 1, Moderately Resistant; 2, Moderately Susceptible; 3, Highly Susceptible).

Table S2. Relative accumulation (RA) and standard error (SE) of CGMMV in *C. melo* and in four wild *Cucumis* species inoculated with the European and Asian isolate.

	European				Asian			
	14 dpi		21 dpi		14 dpi		21 dpi	
**Accession ID**	**Mean RA [1]**	**SE**	**Mean RA**	**SE**	**Mean RA**	**SE**	**Mean RA**	**SE**
BGV004884, Rochet,Ibericus, Spain	6,83E+06	2,50E+07	2,37E+03	1,31E+03	1,56E+04	1,27E+04	8,10E+03	4,38E+03
PI 420176 (Ginsen makuwa) Makuwa, Japan	1,02E+04	5,63E+03	1,97E+05	1,28E+05	1,67E+03	3,55E+03	1,72E+03	3,33E+03
Birjucekutskaja * (Am-BirUkr) Ameri, Ukraine	5,96E+04	5,34E+03	6,06E+04	5,62E+03	6,91E+02	6,10E+02	7,01E+02	6,02E+02
PI 314427 (Koljoznitza) Ameri, Georgia	1,46E+06	1,27E+06	1,75E+06	1,65E+06	not done	not done	not done	not done
PI 185111 (15591) Wild Agrestis, Ghana	3,11E+05	1,28E+05	5,60E+05	4,30E+05	1,12E+03	8,44E+02	1,84E+08	2,08E+08
Ouzbeque * (Am-OuzUzb2) Ameri, Uzbekistan	2,39E+05	1,78E+05	2,53E+05	1,83E+05	3,75E+05	1,56E+05	3,30E+08	8,64E+08
PI 161375 (Songwhan Charmi) Chinensis, Korea	4,25E+05	4,39E+06	1,02E+03	8,02E+02	1,92E+03	1,26E+03	5,76E+04	4,29E+04
PI 273438 (Queen Annes Pocket Melon) Dudaim, Georgia	1,68E+04	1,07E+04	1,74E+04	1,11E+04	4,44E+04	2,69E+04	2,60E+06	3,36E+06
Shiro Uri Okayama* (Con-ShiroJa) Conomon, Japan	1,31E+03	3,93E+02	1,66E+03	1,22E+04	1,55E+05	2,52E+05	5,12E+04	3,75E+03
PI 124112 (2564) Momordica, India	1,87E+04	5,18E+03	2,02E+04	5,57E+03	2,85E+03	7,23E+02	3,00E+03	7,04E+02
BGV 016451 (Amarillo Groc) Ibericus, Spain	6,59E+00	6,17E+00	6,10E+01	1,05E+01	2,55E+07	4,16E+07	2,63E+07	4,03E+07
BGV015753 (Blanco) Ibericus, Spain	3,16E+09	2,13E+09	1,27E+07	1,08E+07	not done	not done	not done	not done
BGV013188 (Pipa de Oro) Ibericus, Spain	3,37E+07	2,40E+07	3,59E+07	2,52E+07	5,90E+06	7,81E+06	5,90E+06	7,81E+06
Asli (In-AsliTun) Ameri Tunisia	2,31E+02	1,31E+01	2,85E+02	1,51E+01	1,15E+01	9,05E+00	8,45E+01	2,21E+01
BGV003686 (Piñoncillo) Ibericus, Spain	1,59E+06	1,18E+06	1,80E+06	1,31E+06	4,69E+05	2,87E+05	4,80E+05	2,92E+05
BGV003692 (Blanco Redondo) Ibericus, Spain	1,10E+08	1,70E+08	1,60E+08	1,30E+08	3,95E+07	1,92E+07	3,99E+07	1,84E+07
BGV003718 (Mochuelo) Ibericus, Spain	2,40E+07	1,50E+07	2,90E+07	1,76E+07	3,51E+05	1,19E+06	3,63E+05	1,02E+06
BGV001365 (Tokash) Ameri, Tajikistan	1,15E+07	1,09E+07	2,14E+04	2,69E+05	1,74E+06	3,44E+06	1,83E+06	3,26E+06
BGV001632 (Kizil-uruk) Ameri, Uzbekistan	5,65E+06	3,59E+06	5,84E+06	3,72E+06	1,36E+04	1,87E+03	1,45E+04	1,21E+03
BGV004871 (Tendral) Ibericus, Spain	9,99E+07	1,62E+08	9,99E+07	1,62E+08	1,06E+05	5,33E+04	1,18E+05	5,38E+04
PI 164493, kakru, Kachri, India	6,50E+04	5,85E+04	6,72E+04	5,93E+04	5,25E+05	4,62E+05	1,30E+05	6,65E+04
PI 164797, 9227, wild agrestis, India	1,67E+06	9,47E+05	1,75E+06	9,52E+05	1,43E+05	2,48E+05	1,88E+05	2,21E+05
PI 180280, Kahkri, Momordica, India	6,42E+04	2,33E+04	6,73E+05	2,43E+04	1,32E+05	1,56E+05	1,56E+05	1,18E+05
PI 381781,Sm1, Momordica, India	8,46E+03	8,16E+03	4,31E+03	1,72E+04	2,63E+05	8,71E+04	2,77E+05	8,55E+04
PI 532839, chibbar, Wild agrestis, India	1,07E+02	4,26E+01	1,11E+02	5,26E+01	2,66E+04	7,05E+03	1,61E+06	8,88E+05
PI 536476, KLM 1733, Maldives	7,93E+04	4,48E+04	8,13E+04	4,73E+04	6,34E+04	7,63E+03	6,43E+04	7,75E+03
PI 614521, KSM 531, Kachri, India	not done	not done	not done	not done	1,92E+03	8,09E+02	2,02E+03	7,01E+02
PI 271332, Khira, Landrace, India	5,04E+04	7,11E+04	5,50E+05	1,31E+05	5,24E+07	3,38E+07	5,05E+05	1,24E+06

BGV004853, Alficos, Flexuosus, Spain	2,68E+03	8,38E+03	8,42E+03	2,67E+03	2,22E+07	5,08E+06	7,97E+05	1,40E+05
PI 381789 (Sm9) Momordica, India	9,82E+06	1,04E+06	6,86E+05	7,51E+04	5,20E+02	9,27E+01	1,09E+04	4,92E+03
PI 169305, Kirkagac, cassaba, Turkey	9,28E+02	4,51E+02	3,55E+03	7,92E+03	5,71E+02	5,86E+02	5,85E+02	5,21E+02
PI 476342, Imljskaha, Ameri, Kazakhstan	3,27E+05	1,71E+05	negative	negative	2,76E+05	3,73E+05	2,52E+05	3,41E+05
BGV001367, Nanatri, Ameri, Georgia	2,63E+08	1,73E+08	4,37E+03	8,05E+05	1,16E+02	7,05E+01	1,25E+02	7,00E+01
La-OgenBul, Dvash Ha Ogen, Cantalupensis, Bulgaria	1,40E+02	2,74E+01	2,18E+04	1,35E+04	3,99E+05	1,62E+05	4,01E+05	1,47E+05
In-HamiChi, HamiMelon, Landrace, China	5,97E+07	7,10E+07	3,50E+02	1,12E+03	1,60E+07	5,52E+06	1,72E+07	5,25E+06
Can-NOFran , Nantais Oblong, Cantalupensis, France	8,68E+00	2,02E+01	5,40E+02	2,23E+02	2,86E+04	1,96E+04	2,96E+04	1,75E+04
Can-NYIsr, Noy Israel, Cantalupensis, Israel	7,21E+04	1,48E+05	2,26E+02	3,65E+01	4,34E+04	2,27E+04	2,86E+04	1,07E+04
Ames 26811, PMR-45, Reticulatus, USA	9,69E+01	9,07E+01	4,15E+04	4,48E+05	4,14E+06	1,00E+06	4,27E+06	1,01E+06
Am-SarakIran , Sarakhs, Kachri, Iran	negative	negative	7,61E+02	2,87E+02	2,81E+04	1,25E+04	5,72E+01	3,99E+02
Am-SouiMor, Souilah, Landrace, Morocco	4,96E+05	3,15E+05	9,69E+01	3,32E+01	6,75E+04	4,47E+04	3,19E+04	2,13E+04
PI 125951, 3584, Ameri, Afghanistan	8,07E+04	1,93E+04	8,71E+02	2,34E+03	2,08E+05	6,44E+04	2,15E+05	6,22E+04
Can-VedFran, Vedrantais, Cantalupenis, France	9,95E+03	5,35E+03	1,18E+01	7,21E+00	2,44E+05	3,12E+05	5,40E+03	3,03E+04
CUM 259, Apelsinaja, Ameri, Russia	9,09E+00	1,58E+00	2,03E+02	2,18E+03	6,04E+06	3,11E+06	6,12E+06	3,03E+06
PI 276660, VIR610-Chandalak, Chandalak, Afghanistan	1,52E+05	4,64E+04	negative	negative	6,88E+00	6,02E+00	1,18E+03	3,24E+02
PI 169331, Altimbas, Ameri, Turkey	2,71E+04	1,18E+04	9,27E+01	2,06E+03	5,41E+03	2,70E+03	5,41E+03	2,70E+03
PI 506459, Salgirskaja Ameri, Ukraine	2,77E+04	4,72E+03	1,10E+04	3,40E+04	1,10E+02	2,15E+02	4,65E+04	1,80E+04
Freeman's Cucumber (Con-FreeCJa) Conomon, Japan	7,44E+02	2,04E+02	5,07E+01	4,39E+01	1,92E+02	2,46E+00	negative	negative
BGV012795, *C. anguria*	negative	negative	negative	negative	negative	negative	negative	negative
BGV012786, *C. ficifolius*	negative	negative	negative	negative	negative	negative	negative	negative
BGV011135, *C.metuliferus*	6,85E+05	8,01E+05	2,48E+06	9,11E+06	1,97E+04	6,86E+04	2,02E+04	5,86E+04
BGV008535, *C.myriocarpus*	8,72E+05	1,06E+06	4,54E+07	6,87E+07	1,43E+04	9,90E+03	1,55E+04	8,90E+03

[1] All data of relative quantification of CGMMV RNA was obtained from 2 biological replicates of 5 pooled plants each, and three technical replicates.
